# Chronic Myeloid Leukemia in a Young Adult

**DOI:** 10.7759/cureus.81220

**Published:** 2025-03-26

**Authors:** Kalan Patel, Jawhar Syed, Ashwin Jagadish, Venkata Vedantam, Daniel Daugherty

**Affiliations:** 1 Internal Medicine, East Tennessee State University James H. Quillen College of Medicine, Johnson City, USA

**Keywords:** adolescent and young adults, bcr-abl1, chronic myeloid leukemia (cml), fluorescence in situ hybridization (fish), myeloproliferative

## Abstract

Chronic myeloid leukemia (CML) is a hematological malignancy commonly affecting older adults. This unusual case involves a 29-year-old male who presented with significant leukocytosis and thrombocytosis. He was later found to possess a mutation resulting in the fusion of the Abelson murine leukemia (ABL1) gene with the breakpoint cluster gene, resulting in the BCR-ABL1 translocation gene. This is also known as the Philadelphia chromosome and predisposed him to the malignancy. This atypical presentation highlights the importance of considering CML in younger patients, broadening differential diagnoses. Understanding such rare but possible occurrences provides insight into patient-centered treatment plans and potentially curative therapies specific to adolescents and young adults.

## Introduction

Chronic myeloid leukemia (CML) is a myeloproliferative neoplasm that accounts for approximately 15% of newly diagnosed cases of leukemia in adults [1]. About 50% of patients are asymptomatic and are often diagnosed through routine physical examinations or blood tests [1]. The pathogenesis of this malignancy stems from a fusion of the Abelson murine leukemia (ABL1) gene on chromosome 9 with the breakpoint cluster gene (BCR) on chromosome 22, resulting in the BCR-ABL1 oncogene that promotes unregulated cellular growth and replication [1]. While CML is most frequently diagnosed in individuals in their sixties, 10% of cases involve those less than thirty years of age [1]. CML predominantly affects older adults, with a median age of onset around 60-65 years. In young adults under the age of 30, CML is rare, with an incidence estimated to be approximately 0.5 to one case per 100,000 population per year [1].

The presentation and response to treatment of CML in young adults and adolescents differ from those in older adults, with younger patients often presenting with higher leukocyte counts and more pronounced organomegaly. Tyrosine kinase inhibitors (TKIs), the mainstay of CML treatment, have transformed the prognosis of this disease, offering excellent cytogenetic and molecular responses across all age groups. Early initiation and adherence to TKI therapy are particularly crucial in younger patients, given their potential for long-term disease control and improved quality of life [2]. This case highlights a rare occurrence of CML in a 29-year-old male, detailing his presentation, diagnostic work-up, and initial response to therapy.

## Case presentation

A 29-year-old male with no significant medical history presented to the emergency department (ED) due to chest pain, cough, rash, and recent abnormal labs. The patient was evaluated at an urgent care for his rash and cough, which had been ongoing for two months. The rash was presumed to be a ringworm infection, and labs were done before the patient was discharged from urgent care with clotrimazole cream. The patient was notified that he had “abnormal labs” later that day and was recommended to go to the ED. Upon arrival at the ED, the patient had a blood pressure of 118/78 mmHg, a heart rate of 79 beats per minute, and a respiratory rate of 15 breaths per minute. He endorsed occasional chest pain, chronic cough, night sweats, intentional weight loss over the past 1.5 years, and chronic fatigue. He denied fever, dyspnea, nausea, vomiting, diarrhea, and constipation. He reported feeling well overall and presented to the emergency department only at the recommendation of providers from urgent care. The patient’s family history was significant for his grandmother, who passed away from an unspecified type of leukemia thirty years prior. The patient denied taking any supplements, over-the-counter medications, or drugs. The patient’s only surgery was a cholecystectomy.

**Figure 1:**
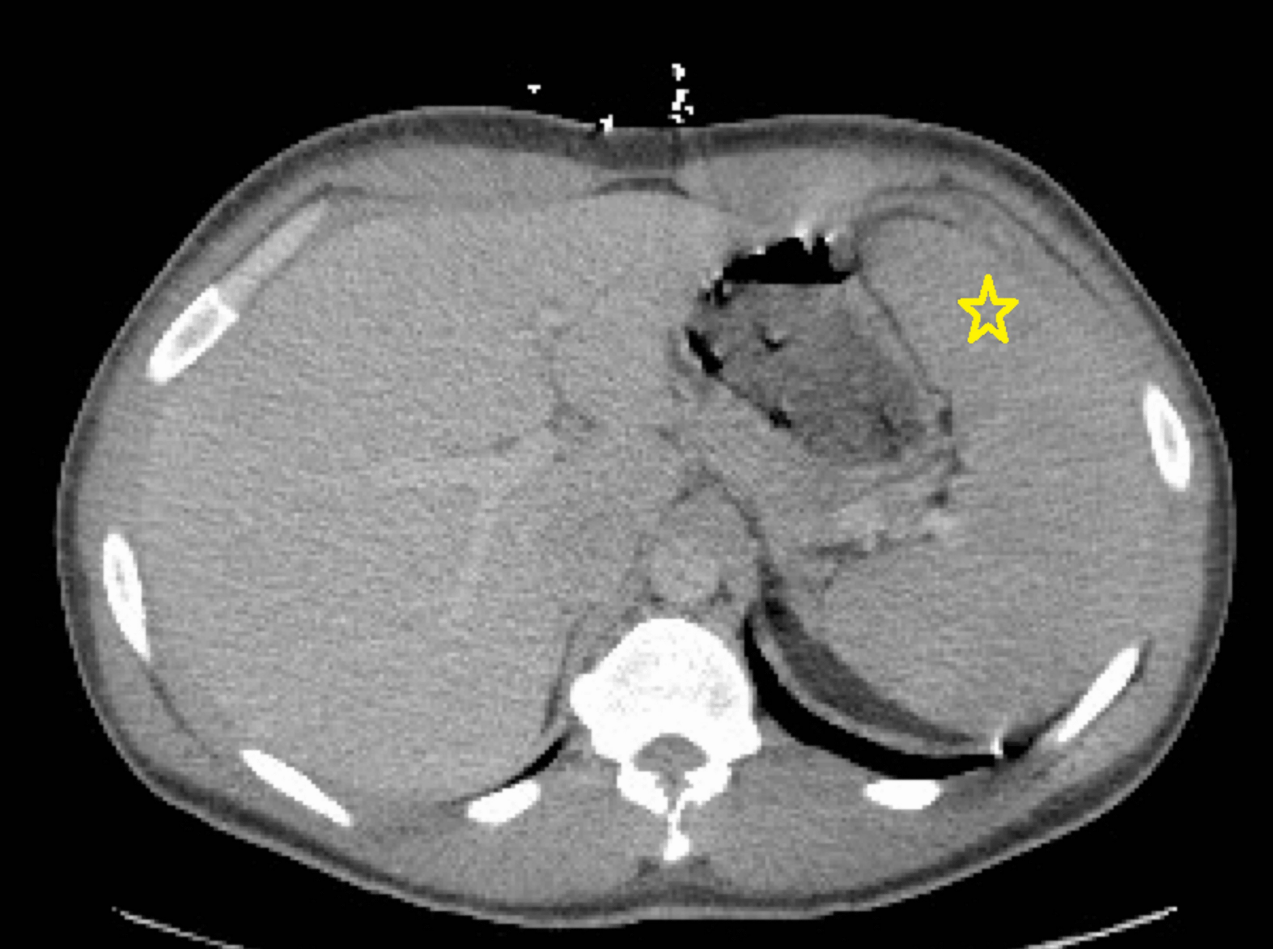
Computed tomography demonstrating splenomegaly The star indicates the spleen.

The patient’s initial labs showed a significantly elevated leukocyte and platelet count, reduced hemoglobin, and significant increases in segmented neutrophils, monocytes, eosinophils, basophils, bands, and blasts (Table 1). Another notable finding was a lactate dehydrogenase level of 628 U/L. His physical exam was notable for an erythematous, ring-like rash on the lower extremities and buttocks, consistent with tinea corporis infection that had improved with clotrimazole cream. Computed tomography (CT) angiogram of the chest, abdomen, and pelvis showed splenomegaly greater than 15 cm (Figure 1). Peripheral blood smears and BCR-ABL1 fluorescence in situ hybridization (FISH) genetic testing were ordered out of concern for an underlying myeloproliferative disorder.

**Table 1:** Complete blood count (H) indicates values that are higher than the reference range. (L) indicates values that are lower than the reference range.

Test name	Result	Reference range
White blood cells (K/uL)	103.1 (H)	4.0-11.0
Red blood cells (M/uL)	3.58 (L)	4.7-6.1 (Males), 4.2-5.4 (Females)
Hemoglobin (g/dL)	11 (L)	13.8-17.2 (Males), 12.1-15.1 (Females)
Hematocrit (%)	33.3 (L)	40-52 (Males), 36-48 (Females)
Mean corpuscular volume (fL)	93	80-100
Mean corpuscular hemoglobin (pg)	30.7	27-33
Mean corpuscular hemoglobin concentration (g/dL)	33	32-36
Red blood cell distribution width (%)	16.4 (H)	11.5-14.5
Platelet count (K/uL)	1,351 (H)	150-450
Mean platelet volume (fL)	9.3	7.4-10.4
Segmented neutrophils (K/uL)	21.65 (H)	1.5-8.0
Lymphocytes (K/uL)	3.09	1.0-4.0
Monocytes (K/uL)	3.09 (H)	0.2-0.8
Eosinophils (K/uL)	4.12 (H)	0.0-0.5
Basophils (K/uL)	11.34 (H)	0.0-0.2
Bands (K/uL)	25.78 (H)	0.0-0.7
Metamyelocytes (K/uL)	10.31 (H)	0.0-0.1
Myelocytes (K/uL)	18.56 (H)	0.0-0.1
Promyelocytes (K/uL)	-	0
Blasts (K/uL)	5.16 (H)	0
Segmented neutrophils (%)	21	40-70
Lymphocytes (%)	3	20-45
Monocytes (%)	3	2-10
Eosinophils (%)	4	1-6
Basophils (%)	11	0-2
Bands (%)	25	0-5
Metamyelocytes (%)	10	0
Myelocytes (%)	18	0
Promyelocytes (%)	-	0
Blasts (%)	5	0

**Figure 2:**
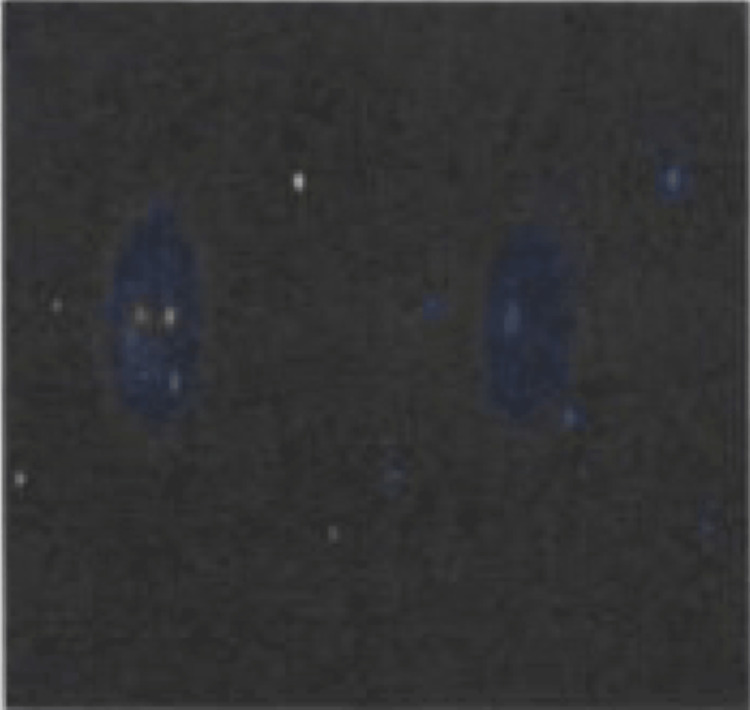
Fluorescence in situ hybridization results

Peripheral blood smears showed marked leukocytosis secondary to granulocytosis with a left shift, mild normocytic/normochromic anemia, and marked thrombocytosis. BCR-ABL1 FISH testing yielded positive results, with chromosome 9 and 22 translocations evident in 87% of cells (Figure 2). Subsequent bone marrow biopsy from the left posterior iliac crest supported a diagnosis of CML. 

While awaiting testing, the patient was given intravenous fluids and started on allopurinol 300 mg daily out of concern for possible tumor lysis syndrome. Once the patient underwent a bone marrow biopsy, he was also started on hydroxyurea 500 mg twice daily to lower the tumor burden. The patient was discharged on the aforementioned dosages of allopurinol and hydroxyurea until follow-up.

The patient had an uneventful two-day hospital course, alongside a reduction in leukocytosis from 103,000 K/uL on admission to 81,000 K/uL at the time of discharge. Once inpatient testing was complete, the patient was scheduled for a one-week outpatient appointment with hematology and oncology for further discussion and management.

## Discussion

CML is a myeloproliferative neoplasm characterized by the presence of the BCR-ABL1 fusion gene or Philadelphia chromosome. It typically presents with granulocytic proliferation, left-shifted maturation, and occasional increases in blasts. CML is clinically classified into three phases, namely, chronic phase (CP), accelerated phase, and blast phase, with prognosis worsening in the latter stages [2].

CML can manifest across all age groups, but its incidence increases with age. In Western countries, the median age at diagnosis is approximately 64 years [3], while clinical studies have reported a slightly younger median age of around 54 years [4]. Historically, before the advent of imatinib, older age was considered a negative prognostic factor, as reflected in the Sokal and Euro risk stratification scores [5]. However, with the introduction of tyrosine kinase inhibitors like imatinib, the overall prognosis has significantly improved, and the negative impact of advanced age has diminished [6]. A study comparing patients below and above 65 years of age treated with imatinib found no significant differences in outcomes between the two age groups [7]. 

Interestingly, adolescents and young adults (AYAs), defined as those aged 15-29 years, demonstrate unique disease characteristics and responses to treatment. A study comparing AYAs with patients over 29 years of age diagnosed with CP-CML found inferior response rates in AYAs treated with first-line TKIs [8]. This observation aligns with findings in other malignancies, such as acute lymphoblastic leukemia (ALL) [9], breast cancer [10], colorectal cancer [11], and soft tissue sarcomas [12], where AYAs exhibit distinct biological features and, in some cases, worse prognoses [13].

In CML, AYAs often present with more aggressive disease features, including elevated white blood cell (WBC) counts, increased blasts in peripheral blood, lower hemoglobin levels, larger spleen size, and more frequent organomegaly-related symptoms [8]. One study also reported significantly lower cytogenetic and molecular responses in AYAs [8]. Notably, a more significant proportion of younger patients exhibited BCR-ABL1 transcript levels above 10% at three months compared to patients older than 44; such transcript levels are associated with unfavorable prognosis [14,15] and necessitate closer monitoring [16].

These findings suggest potential differences in the underlying disease biology of CML in younger patients. Similar age-related genetic and biological differences have been identified in other cancers. For instance, younger patients with colorectal cancer show increased chromosomal instability and more significant tumor invasion [17]. In contrast, younger individuals with papillary thyroid cancer exhibit distinct gene expression patterns correlating with advanced presentation but better prognosis [18]. In breast cancer, unique genetic pathways in younger women are recognized as negative prognostic markers. Similarly, in ALL, distinct biological features in AYAs, such as higher prevalence of the Philadelphia chromosome, intrachromosomal amplification of chromosome 21, increased promoter methylation, and more frequent T-cell immunophenotypes, contribute to poorer outcomes [9].

The potential involvement of differences in stem cell biology, bone marrow microenvironment, or signaling pathways in AYAs with CML remains an area for further research. While BCR-ABL triggers multiple pathways that are effectively inhibited by TKIs [19], data specific to AYAs are lacking.

Another possible explanation for the more aggressive disease presentation in AYAs could be delayed diagnosis. Younger patients may seek medical attention less frequently than older individuals, resulting in diagnosis only when symptoms become apparent. However, this hypothesis remains speculative due to the lack of supporting data [9].

A German study concluded that AYAs with CML show features of a more aggressive disease, indicating possible biological differences of younger patients that need to be investigated. Moreover, the higher transcript level at three months suggests a worse prognosis of AYAs. Nevertheless, younger patients do well in spite of poorer prognostic indicators [20].

## Conclusions

This case underscores the atypical presentation of CML in a 29-year-old patient, a demographic where this malignancy is infrequent. The patient's significant leukocytosis, thrombocytosis, and BCR-ABL1 translocation exemplify the aggressive disease features often seen in AYAs. Despite these aggressive presentations, AYAs typically exhibit a robust response to TKIs, emphasizing the critical importance of early diagnosis and timely initiation of therapy.

Furthermore, the unique biological and genetic characteristics observed in CML among AYAs highlight the need for a tailored approach to diagnosis, risk stratification, and treatment. This case reinforces the importance of including CML in the differential diagnosis for younger patients presenting with persistent leukocytosis or related symptoms, broadening the scope of clinical vigilance. Continued research into the biological underpinnings and treatment responses of AYAs with CML is necessary to optimize outcomes and advance patient-centered care in this subgroup.
